# inproTeamED - clinical reasoning in the emergency department as an individual and interprofessional process: a qualitative analysis

**DOI:** 10.1186/s12873-026-01827-x

**Published:** 2026-10-30

**Authors:** Rena Isabel Amelung

**Affiliations:** Department of Health, Hochschule Bielefeld, University of Applied Sciences and Arts, Interaktion 1, 33619 Bielefeld, Germany

**Keywords:** Clinical assessment, Clinical reasoning, Interprofessional collaboration, Emergency department, Interprofessional communication

## Abstract

**Background and objective:**

Patients in the emergency department (ED) are assessed and treated by the ED-Team consisting of nurses and physicians. The clinical assessment (CA) of emergency patients and the clinical reasoning (CR) of both professions play a decisive role in determining the quality of emergency care. This pilot study explores CR in daily routine practice of the ED on a cognitive-individual level, relying on the Dual-process Theory and on a social-interprofessional level, arguing interprofessional collaboration (IPC) as a part of CR. At least, organizational-structural factors influencing CR are analyzed as a third level.

**Methods:**

This is a qualitative study of 13 semi-structured individual interviews with nurses and physicians from three EDs in Germany, analysed using content analysis and methodological fundation.

**Results:**

In the individual-cognitive CR, both physicians and nurses groups identified similar key factors for CA and used Systems 1 and 2 of the Dual-process Theory in their CR. On the social-interprofessional level, IPC was generally described positively, naming characteristics of good IPC and communication barriers were reported as rare. As possible organizational-structural factors influencing CR working experience as a prerequisite, size and region of the ED, age structure of the team and digitization were found.

**Conclusion:**

This pilot study provides insights into the CR of nurses and physicians in the ED and analyzes CR on an individual-cognitive, a social-interprofessional and an organizational-structural level, allowing to conclusions regarding the prerequisites for successful ED-teams.

## Background

In emergency departments (ED), many patients are treated daily by nurses and physicians. In order to provide good care for emergency patients, they must be correctly assessed within a short period of time, a preliminary diagnosis must be determined and appropriate measures must be derived. The two professions of nurses and physicians work closely together to ensure the best possible outcomes for their emergency patients.

The clinical assessment (CA) of emergency patients includes the systematic collection, structuring, weighting and initial interpretation of relevant information from the patient’s history, presenting symptoms, vital signs, physical examination, assessments and diagnostic procedures such as laboratory results and imaging to evaluate the urgency of treatment through triage, the patient´s clinical condition, acuity and potential diagnosis to guide subsequent clinical decisions [1, 2]. In EDs, the volume of emergency patients is often high, and the complexity of case scenarios is steadily increasing. Based on the CA by the emergency nurses and physicians, prioritization is carried out and decisions regarding interventions are made [3, 4].

The CA of emergency patients as an interprofessional process extends above their stay in the ED and goes significantly beyond triage, which aims to quickly sort patients upon arrival at the emergency department in order to determine the priority for further treatment and to ensure the right person receives the right resources, in the right place, at the right time [5]. Studies in this setting primarily analyze the use of triage through guided systems such as the Manchester Triage System (MTS) as part of the CA [6], are outcome-oriented [7], or examine high-stress situations such as trauma room management or resuscitation scenarios [8], while less frequently addressing day-to-day collaboration in emergency settings.

But assessing emergency patients goes beyond simply collecting and organizing information according to fixed schemes such as MTS and admission forms. The perception of the patient as a whole together with their environment - a “clinical eye” - based on the intuitive recognition of patterns and several more cognitive processes are required.

Depending on the source consulted, CA can be understood as a component of or a prerequisite for effective clinical reasoning (CR), or it could be understood as a synonym for it - if it refers to more than data collection but also encompasses the cognitive processes involved in decision-making, diagnosis and planning of interventions. In any case, the two concepts are closely linked, and there is no uniform definition of CA and CR that would allow for a clear distinction between them [9–11]. In addition, there are differences in the understanding of CR among the various professional groups in patient care [11]. In this study, CA is understood as a component of CR, distinguishing the concepts referring to the cognitive processes of CR which include interpreting and integrating relevant information and formulate clinical judgements und guide decisions.

Generally, CR is understood as the cognitive process of assessing patients, recognizing patterns, building diagnostic hypothesis and enabling decision-making [9, 12, 13] as a core competency of physicians [14, 15].

Since assessing emergency patients and making decisions in the emergency department often involve time pressure and incomplete information—and thus a great deal of uncertainty—the information must be quickly evaluated based on experience and, when necessary, systematically verified. Although theoretical models cannot fully capture the cognitive processes and complexity of decision-making in real clinical situations, they can provide a helpful framework for understanding them. The Dual-Process Theory [16] offers a theoretical model and framework that captures both the experience-based, rapid aspect and the systematic, reliable aspect of the decision-making process, making it suitable for examining these complementary modes of information processing. This theory of CR is currently the dominant model in the ED as a universal approach to clinical decision-making by physicians. It posits two decision-making systems. System 1 is an intuitive approach, based on pattern recognition and depending heavily on the decision-maker’s experience. System 1 relies on heuristics and inductive reasoning, it is fast, efficient, and requires little effort. Decisions made on the basis of System 1 are poorly reflected upon, context-dependent, influenced by various factors, and strongly determined by the decision-maker’s working experience. The System 2 approach, on the other hand, is analytical, based on hypothesis testing and deductive reasoning; it is slow and resource-intensive, but also less prone to error [16]. Pellacia et al. assume, that a harmonious interaction between Systems 1 and 2 both driven by underlying knowledge could be the crucial key function of CR in the ED setting [17]. A dialectic between intuitive and analytical cognition throughout the individual CR process with first predominantly intuitive impressions, followed by validation through specific analytical strategies and reassurance strategies in cases of remaining uncertainty has been discussed before [18].

The CR as the process of generation and ordering of diagnostic hypotheses also includes processing environmental information and linking them to experiences and knowledge which is based on working experience [14]. Particularly the intuitive System 1 is depending on working experience and context. The required quick decision-making based on incomplete information, huge number of interruptions and high stress level in the ED would be typical influencing factors [16]. Despite this known situated activity of CR, strongly shaped by the context in which it occurs, and the contextual specificities of the ED, there are less studies centering CR in the ED than expected. In particular studies on the cognitive processes of CR in the ED and influencing factors are scarce or show conflicting results [15].

Recent studies have demonstrated similar cognitive processes among registered ED nurses when assessing emergency patients as found among physicians [19], CR representing also a core competence of nurses [4, 10]. Nurses’ CR enhances the quality and safety of care and contributes to improved health outcomes [4]. CR among registered nurses in ED involves assessing complex problems, performing triage decisions, anticipating complications, intervening effectively and preventing the deterioration of patients’ clinical conditions, which significantly influences treatment outcomes [4, 19]. In their initial CA, nurses go beyond triage algorithms to enable a comprehensive, individualized, and holistic perspective on the urgency of treatment and to guide the care process. In doing so, they not only utilize fixed algorithms such as MTS reflecting System 2 of the Dual-process Theory of CR, but also holistic reasoning as in System 1, relying on knowledge and experience and including the whole situation in the ED [19].

However, CA and the derivation and implementation of therapeutic measures are not solely based on the personal actions of individuals. Especially in the ED setting, the interprofessional collaboration (IPC) of the ED team plays a crucial role in the successful care of emergency patients and can be considered as CR on a social-interprofessional level. Therefore, cognitive decision-making processes, as described in the Dual-Process Theory, should not be considered in isolation within a single individual; rather, the exchange of information among the professional groups that assess patients and make decisions should be taken into account.

In EDs, close IPC between the nurses and physicians is absolutely essential and a key factor in increasing effectiveness [20]. The benefits of effective IPC are well-documented and include an integration of different professional perspectives and expertise, a greater job satisfaction and engagement while reducing stress, an improvement of communication, coordination, efficiency and quality of patient care [7, 21]. Nevertheless, barriers as divergent values and beliefs, imbalances of authority, limited understanding and conflicting perceptions of each profession’s role and responsibilities between nurses and physicians are described [7, 22]. In turn, the quality of IPC significantly influences patient safety and team satisfaction [23].

IPC is a complex and dynamic process that can vary in intensity [20]. The increasing complexity of health issues among patients inevitably makes professionals interdependent; collaboration is essential to ensure high-quality healthcare. Sharing responsibilities and decision-making, especially in a dynamic working field like the ED, requires joint action directed toward a common goal as the best treatment for emergency patients. Effective interprofessional teams and functioning ICP are grounded in a collegial relationship characterized by principles of good collaboration including open and honest communication, mutual trust and respect, appreciation and common values, mutual understanding and respect for each other’s professional roles, a balance of power, flat hierarchies and an awareness of each member’s expertise [20].

Functioning ICP requires an exchange of information regarding the health status of emergency patients and an analysis of the feasibility of possible options, but also takes into account the influence of the professional roles of individual team members, as well as organizational processes and systemic factors that either support or limit these roles [24].

Because finally, systemic factors and organizational metrics also influence CR on the individual cognitive level and on the social-interprofessional level.

### Study objective and research questions

In this study, CR in daily routine practice of the ED was examined on an individual-cognitive level and as well as on a social-interprofessional level including IPC and interprofessional communication. Additionally, potentially relevant factors of the organizational-systemic level were analyzed, influencing CR on the individual and interprofessional level.

Following central research questions and subquestions were defined:

1. How do nurses and physicians perform their individual CR in daily routine of the ED?
  - How is CA in general performed, which topics are important? Are there differences between the two professions?
  - Report both nurses and physicians factors in the CA and cognitive actions corresponding to System 1 and 2 of the Dual-process Theory? Are there differences between the two professions?
2. What can be concluded regarding IPC between nurses and physicians in ED as a social-interprofessional CR? How do different professional perspectives help to supplement, challenge, or verify intuitive, experience-based assessments through systematic CR?
  - Where is IPC happening in the daily routine of the ED?
  - Which prerequisites and barriers for functioning IPC in the ED can be reported?
  - Which role play team spirit and communication in IPC? How is communication characterized regarding communication channel, tone, technical language and content? Are there misunderstandings? Do nurses or physicians communicate or talk differently when interacting with the other professional group?
  - Is the individual-cognitive CR discussed within the ED-team? What was the purpose of discussing one’s own CR with the other profession, and what were the consequences?
3. Which organizational-structural factors influence CR on the individual and social level?
  - How important is clinical working experience for the ED, influencing individual-cognitive CR?
  - Which role play interprofessional debriefings and team-timeouts in the communication and IPC as part of the social-interprofessional CR?
  - Does the size or region of ED influence the IPC and social-interprofessional CR?
  - Which impact does digitization have on communication, IPC and social-interprofessional CR? Does age distribution play a role?

## Methods

This qualitative pilot interview study included 13 face-to-face, semi-structured individual interviews with nurses and physicians (10 nurses, three physicians) who were permanently employed at one of the three EDs of Klinikum Bielefeld gGmbH in Germany at the time of the study. This study is part of the inproTeamED project (inproTeamED - Emergency department as a site for collaborative communicative diagnostic processes in the assessment of emergency patients); a further publication from the project is currently being developed, analyzing changes of diagnosis along the patient journey using quantitative data.

The methodology and results are reported in accordance with the COREQ (COnsolidated criteria for REporting Qualitative research) guidelines [25].

### Research team and reflexivity

The research team consisted of individuals with clinical as well as methodological experience in qualitative research. The interviews were conducted and evaluated by an interprofessional team. The research team was deliberately composed of individuals with experience in direct patient care and emergency room settings. Compiling a research team with diverse backgrounds (physician and nurses) was tried to maintain researcher triangulation to reduce biases.

The project leader is a physician with a subspecialty in clinical acute and emergency medicine and many years of experience as a physician in direct patient care and in the ED, with a Doctorate in Medicine (Dr. med., Germany) and Professor for fundamentals of Biomedical Science at the University of Applied Sciences and Arts Bielefeld in Germany. The project leader is experienced in conducting and analyzing qualitative interviews [26].

The three research assistants employed by the project are registered nurses, two of whom work in EDs that were not participating in the study and one works in an intensive care setting while they are also studying at the University of Applied Sciences and Arts Bielefeld in Germany. All members of the research team are female.

All interviews were conducted in the presence of two interviewers of the research team, one person leading the interview, the other listening and sometimes making notes. Most of the interviews were directly conducted by the project leader. To enhance interpretive credibility, the data were independently analyzed by two researchers with different professional backgrounds (physician and nurse) and subsequently discussed by consensus to enable analytical triangulation. During data collection and analysis, regular reflective team meetings were held to reflect on the potential influence of professional perspectives and prior experiences on the interpretation of the results.

The project leader was working in one of the three EDs several years before the interviews were conducted. Because of previous work at one of the three participating EDs, a relationship had been established with some of the interviewees prior to study commencement. Some of the participants knew the project leader as a former colleague and physician, but they did not know anything about the personal goals or reasons for doing the research. At the time of the study, none of the researchers had a direct supervisory relationship with the participants or were employed in any of the EDs that contributed to the study. Furthermore, the participants were not informed in advance of any assumptions, motivations, or interests that the members of the research team might have regarding the research topic.

### Study design

#### Theoretical framework

The approach followed an interpretive research paradigm with a focus on reconstructive understanding. This approach assumes that reality is not objectively measurable but is interpreted, experienced, and jointly constructed by the participants. The focus is therefore not primarily on measuring frequencies but on understanding meanings, experiences, and decision-making processes. During the interview, an attempt was made to reconstruct the underlying cognitive, decision-making, and interaction processes in order to comprehend them. The Dual-Process Theory of CR and the concept of IPC formed methodological orientation of the interpretational analysis.

#### Participant selection

For recruitment, participants were purposively selected. Following consultation with the research department of Klinikum Bielefeld GmbH in Germany, contact was established with the chief physician of the three emergency departments, as well as with the nursing department heads and team leaders. Possible participants were approached face-to-face in an informational event and a contact list was created for interested staff members to sign up for. We contacted interested individuals via email and scheduled an interview. A reminder email was sent three days before the scheduled interview.

The inclusion criteria for participant selection are documented in Table 1. Recruiting a sample that was as heterogeneous as possible proved difficult. After giving consent to the interview, no participant withdrew their consent or dropped out. A total of 13 interviews were conducted, 10 of which were with nursing staff and three with physicians. Due to the staffing composition of the three included emergency departments and the decision to include only permanently employed staff in the emergency department, there was a significantly higher proportion of women and a lower proportion of physicians among the interviewees; thus, the results should primarily be understood from the perspective of nursing staff with limited input of physician perspective. Overall, the sample reflects the distribution of staff working in the emergency department (significantly more nurses than physicians). A roughly equal distribution was achieved between employees from a large urban and a smaller rural service region of Klinikum Bielefeld gGmbH in Germany.

**Table 1:** Inclusion criteria

Inclusion criteria	• Permanent employment at Klinikum Bielefeld GmbH • Permanent assignment to one of the three central emergency departments of the institution departments (no on-call staff, medical on-call service, rotating resident physicians) • Belonging to the professional group of registered nurses or physicians (no nursing or medical assistants)

#### Setting

The interviews were conducted in a quiet room at each of the three emergency departments’ respective hospital locations. The interview rooms were provided by the Klinikum Bielefeld GmbH in Germany. They were deliberately located a considerable distance from the respective emergency departments to ensure that the interviews could not be overheard by third parties and that there would be no disturbances. All interviews were conducted in the presence of two interviewers of the research team, one person leading the interview, the other listening and sometimes making notes. Most of the interviews were directly conducted by the project leader. No non-participants out of the research team were present.

In total, approximately 80,000 emergency patients are treated annually at the three ED locations of Klinikum Bielefeld GmbH in Germany by interprofessional teams consisting of emergency nurses and emergency department physicians. In the EDs studied, triage is carried out solely by registered emergency nurses and is performed using the scientifically validated MTS, which assigns emergency patients to a priority level based on their symptoms. Therefore, triage was considered as one aspect of the overall clinical assessment performed by emergency nurses.

The characteristics of the sample are shown in Table 2. The characteristics were recorded by having participants fill out a short, written questionnaire by hand before the interview began.

**Table 2:** Sample

Age	Average age 49 years Median 52 years	
	Age groups 18–40 years 41–50 years >50 years	Number of participants 3 3 7
Gender	m = 3; f = 10; *n* = 0	
Occupation	Physicians = 3, a1-a3; Nurses = 10, p1-p10	
Work location	Rural = 7; Urban = 6	
Years of working experience	Years of working experience	Number of participants
	1–5 years	1
	6–15 years	3
	>15 years	9
Years of working experiences in the ED	Years of working experience in the ED	Number of participants
	1–5 years	4
	6–15 years	3
	>15 years	6

Gender: *m* = male, f = female, d = divers

#### Development of the interview guide

The guide was constructed according to the SPSS principle (Collect, Check, Sort, Summarize) as described by Helfferich [27]. First, potential topics were derived from a literature review and a methodological base was established and questions were developed (Collect). Subsequently, questions were reviewed for openness, clarity, and avoidance of leading language (Check) by faculty colleagues of the project leader, experienced in qualitative research, and individuals from the ED´s work environment who were not otherwise involved in the project. The questionnaire was adapted accordingly based on their input.

In the next step, the topics were structured around central research dimensions such as individual CR processes, IPC and team interaction (sorting). Finally, the guide was streamlined and consolidated and again reviewed (subsuming).

The guide was pre-tested in a pilot interview to review the clarity and scope of the questions and their wording, as well as their order and subsequently slightly revised. Since only one question was added, and the relevance of these questions had been identified during the pre-test interview the pre-test interview could be included in the data analysis.

The development of the questionnaire was methodologically based on the concepts of dual-process theory [16] and the concept of interprofessional collaboration [20]. Nevertheless, it was decided to deliberately focus the questions in the questionnaire on practical work and everyday situations in the ED. For example, respondents were asked at which points in the process contact occurs with other professional groups, what form this contact takes, through which communication channels it occurs, and what topics are discussed; they were then asked to describe specific examples of their experiences.

The aim was to ask about personal experiences as naturally as possible, so that the interviewees could recount them from memory, thereby minimizing any influence on their accounts resulting from prior information or discussions regarding the theoretical framework used. Thus, to explore the CR, the term itself was deliberately not used; instead, the interviewee was asked to explain, step by step, how they would proceed when assessing a patient, how they would reach conclusions and determine appropriate measures, and how they would collaborate with other professional groups. Interviewees were asked, if possible, to recount a specific event from the past few days or weeks in order to provide detailed recollections and depict everyday routines as realistically as possible. To probe System 1 of the Dual-Process Theory, the role of gut feeling was specifically addressed, as otherwise respondents might have reported only highly assessment-based or prescribed inquiries and courses of action.

Pre-testing revealed that this additional question was necessary. During the pretesting, the interviewee briefly brought up the topic of intuition on his own, but then dropped it; when prompted, the interviewee provided further details. For this reason, a question about the role of “gut feeling” was added to the questionnaire after the pretesting to ensure that the use of System 1 was not under-explored.

To further examine interprofessional collaboration as a social level of CR, interviewees were asked to describe their interaction with the other professional group in everyday working situations, their communication and the team structure. In addition, systemic and organizational factors, such as the digitization of processes in the ED, were considered. Specifically, the focus was on whether the transition from handwritten notes to documentation in the computer system had changed communication.

#### Data collection

Before conducting the interviews, the characteristics were recorded by having participants fill out a short, written questionnaire by hand before the interview began (Table 2). The participants did not receive any questions, prompts or guides before the interviews. No repeat interviews were carried out. The interviews were digitally audio-recorded from late 2024 to early 2025 and subsequently pseudonymized and transcribed by an external office.

A few field notes were made during and after the interview but were nor included into the analysis. The duration of interviews was estimated to 45 and 90 minutes and varied between 25 and 90 minutes. This variation was inherent to the semi-structured interview approach and the use of open-ended questions, which allows participants to elaborate on topics according to their individual perspectives and experiences. Interviews were not terminated based on a predefined time point, but rather when the relevant topics had been sufficiently explored. Consequently, the length of the interviews reflected differences in participants´ experiences, engagement and level of detail in their responses. Transcripts were not returned to participants for comment.

### Data analysis

#### Method

A qualitative interview study using semi-structured individual interviews was conducted.

The data were analyzed using structured qualitative content analysis based on Mayring’s approach [28]. This method enables a systematic, rule-based qualitative analysis of the interview material and allows for a systematic focus on specific content-related aspects within the material. The interviews were analyzed using the MAXQDA 24 software [29]. The interviews were conducted in German and the analysis was translated into English for publication.

#### Development of the coding tree and coding process

The a priori category system was based on the semistructured interview guide, whose main content areas served as the initial main categories. This structure was tested by first coding of the same two interviews by all members of the research team. Afterward, the code tree was discussed, changes based on the coded interviews were adjusted and refined by the research team to ensure consistent coding and comparability. The deductively developed coding tree was applied to the material, and iteratively adjusted and inductively expanded during the analysis process.

**Fig. 1:**
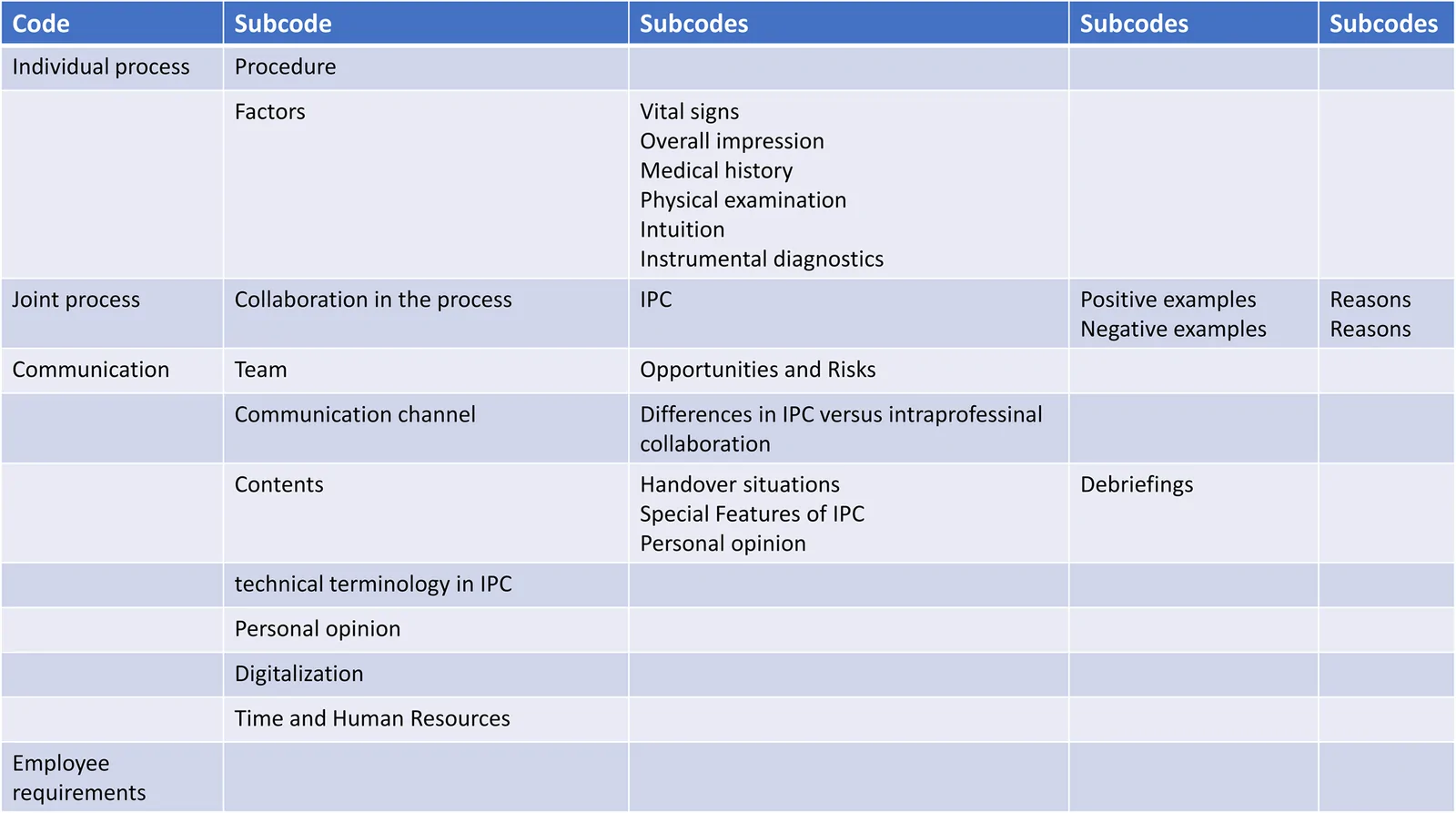
First analytic step: categories relevant for the article

The coding was carried out by four people: the project leader and three research assistants employed by the project. All interviews were distributed among the three research assistants, and each interview was coded by one research assistant and by the project leader. After the two researchers had coded the interviews independently, the respective pairs met and went through the coding point by point, discussed discrepancies, and reached a consensus ensuring intersubjectivity. During the coding process, several additional codes and subcodes were developed inductively and added to the system. Whenever a new code was introduced, the material was reviewed again and consensus was re-established. A description of the code tree of the first analysis step is displayed in Fig. 1.

The final sample comprised 13 interviews. Sample adequacy was assessed iterative during data collection and analysis, considering the information power of the sample, the specificity of the study population and the richness of data. After each interview was analyzed, newly identified codes were documented and compared with the existing code tree. The research team specifically monitored weather subsequent interviews generated new codes, new dimensions of existing themes or substantially different perspectives until the existing themes were considered suffciently rich and well developed to address the research questions (reached after *n* = 10 interviews). Given the limited number of physicians included (*n* = 3), saturation cannot be assumed for the physician subgroup itself, although the same monitoring was done for all interviews, limiting comparability between professional groups and reducing the transferability of the results. Findings related specifically to physicians should therefore be interpreted as exploratory rather than trustworthy.

#### Application of the Methodology and abstraction

In a subsequent analytical step, findings from the interviews relevant to the Dual-Process Theory of CR and the concept of interprofessional collaboration were extracted to facilitate complementary perspectives on CR. In this way, the analysis focuses, on the one hand, on individual CR through the personal acquisition and processing of information, and, on the other hand, on the social-interprofessional level of CR, in order to illustrate how a shared CR is established, exchanged, and coordinated among the professions.

The Dual process Theory was used as a theoretical framework to interpret the findings related to CR at the individual cognitive level. Relevant statements were subsequently assigned to System 1 and /or System 2 according to the characteristics described by Croskerry [16]. Statements reflecting both rapid, experience-based processing and deliberate, analytical reasoning were considered as representing an interplay of both systems. The framework was applied to both professional groups to examine whether and how both modes of processing were reflected in the accounts of nurses and physicians.

**Fig. 2:**
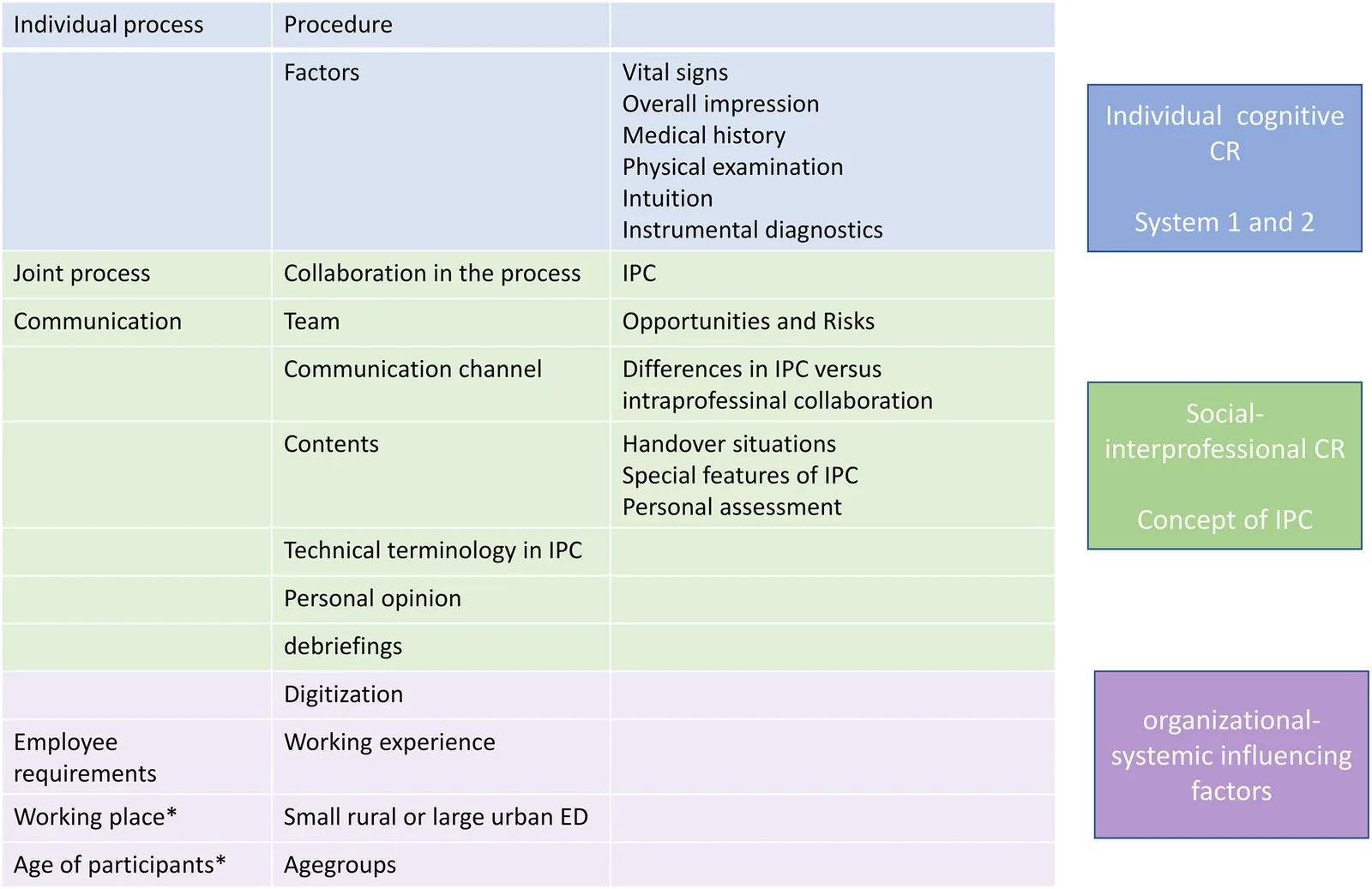
Second analytic step: methodological interpretation and abstraction. * variables from the short questionnaire

For the findings related to IPC, the concept of D’Amour was used [20]. In addition, a targeted search was conducted for the exchange of assessments of emergency patients between professional groups, analyzing the social level of CR. In addition, the factors influencing both individual and interprofessional CR were identified. For the influencing factors, also variables from the short questionnaire—such as the participants’ age and whether they worked in a small rural or a large urban ED—were examined in addition to the categories from the coding. The second analytical step as an abstraction process is shown in Fig. 2.

#### Inclusion of the interviewees to the discussion of results

Once the results were available, the interviewees were invited to a wrap-up session, which was voluntary and independent of their participation in the interview. During this session, which was attended by half of the interviewees, the results were presented and discussed with the group. Field notes were taken for documentation purposes. No contradictions were found between the results and their interpretation and the opinions expressed by the interviewees present. However, since only half of the interviewees were present and the open discussion could have limited the validity of the statements, the results of this group discussion were not included in the present study.

### Ethics and data protection

The interviews described were conducted with the approval of the competent ethics committee of the Westfalen-Lippe Medical Association (Ärztekammer Westfalen-Lippe, file number 2024–316-f-S), in accordance with national law, and in compliance with the Declaration of Helsinki (in its current, revised version). A written informed consent form was available for all participants.

To ensure compliance with data protection regulations, a privacy notice, an information letter, and consent forms for participation in the interviews were developed. Participation in the interviews was voluntary and not tied to working hours. The interview participants were informed of their right to withdraw their consent. The participants’ contact information was stored for the duration of the project, with their consent, to invite them to a closing event featuring a presentation and discussion of the results. This list was separately password-protected and accessible only to the project leadership. It will be deleted upon completion of the project.

Immediately after transcription, the recorded audio files were deleted. To the extent that personal characteristics were recorded in the interview data, these were pseudonymized by the contracted transcription agency.

Codes were assigned to the transcribed interviews to differentiate them and analyze them in groups based on the characteristics collected in the short questionnaire. During the analysis, the codes created could not be personally linked to individual participants.

A master data list was created using the participants’ pseudonymization codes, documenting, among other things, demographic data from the short questionnaire survey, the date of data collection, the status (transcription pending/completed), and the existence of the informed consent form. In all cases, the data initially consisted of the audio recordings and their corresponding interview transcripts. The key data list (with code assignments) was separately password-protected and was accessible only to the project management.

The transcribed interview data was stored and analyzed on a secure drive at Bielefeld University of Applied Sciences and Arts in Germany (Hochschule Bielefeld, HSBI). Access to the drive was restricted to individuals directly involved in the analysis of the interviews.

## Results

### Individual-cognitive level of CR in daily routine of the ED

#### Procedure of the individual CA

Both physicians and nurses of ED considered an unbiased, independent CA to be of great importance. The focus here was on the current clinical picture and its comparison with objective findings, such as the vital signs that have been recorded. Both professional groups cautioned against uncritically accepting interpretations, emphasizing that primary care physicians “often have only very brief contact, sometimes none at all” (a2, item 35). Premature assumptions and categorizations, in the sense of confirmation bias, were also considered problematic. For unstable patients, reassessments and monitoring are conducted until a definitive diagnosis was established.

**Fig. 3:**
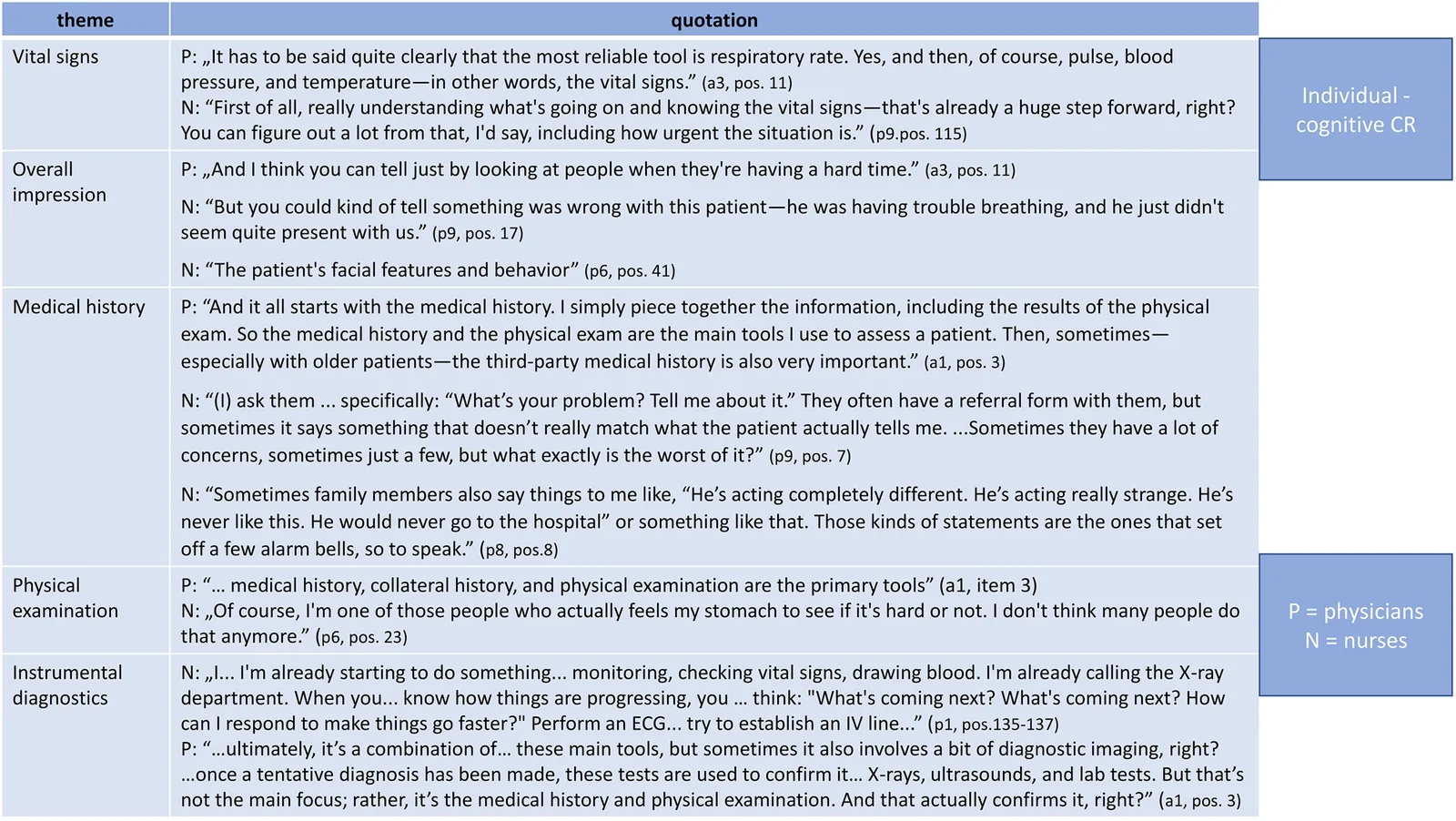
Individual-cognitive CR: themes and quotations

Differences between the professional groups were evident primarily in their perspectives and priorities. From a physician perspective, medical history, collateral history, and physical examination were viewed as “primary tools” (a1, item 3); diagnostic imaging was used to confirm hypotheses or rule out differential diagnoses. Nurses tended to focus on accelerating and harmonizing the process for example by proactively and independently driving forward diagnostic procedures as imaging and blood tests and preparing measures before physicians instructed them to do so and as a result managing patient flow. Experienced nurses in the sample emphasized that the fixed criteria of the MTS alone, although used for triage, are insufficient for assessing patients as a whole and need to be expanded to a more holistic view of the patient and his or her environment like family members and life situation. Physician participants tended to emphasize diagnosis and the selection of interventions. Figure 3 shows quotations corresponding to the main themes within the individual-cognitive CR in the sample.

**Fig. 4:**
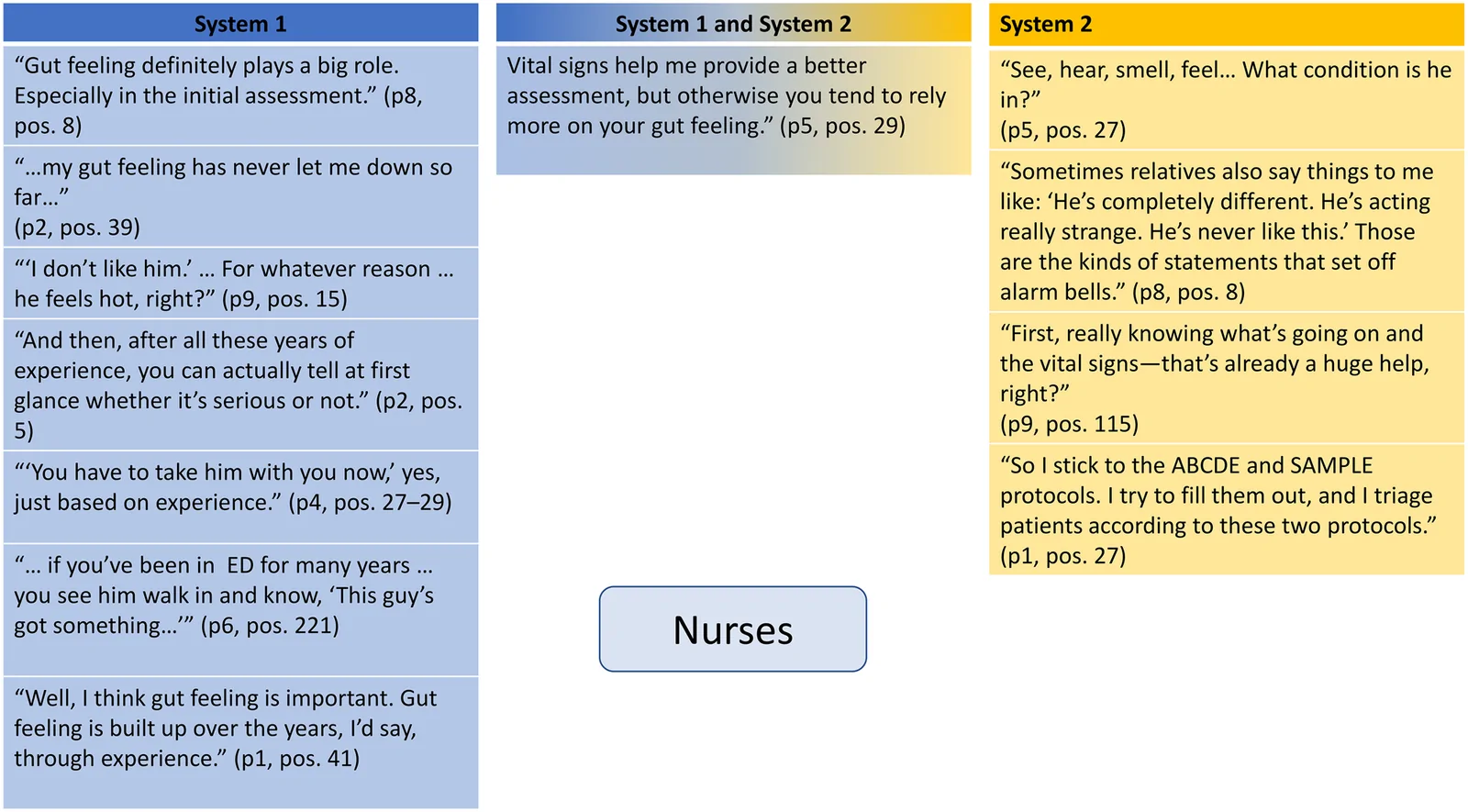

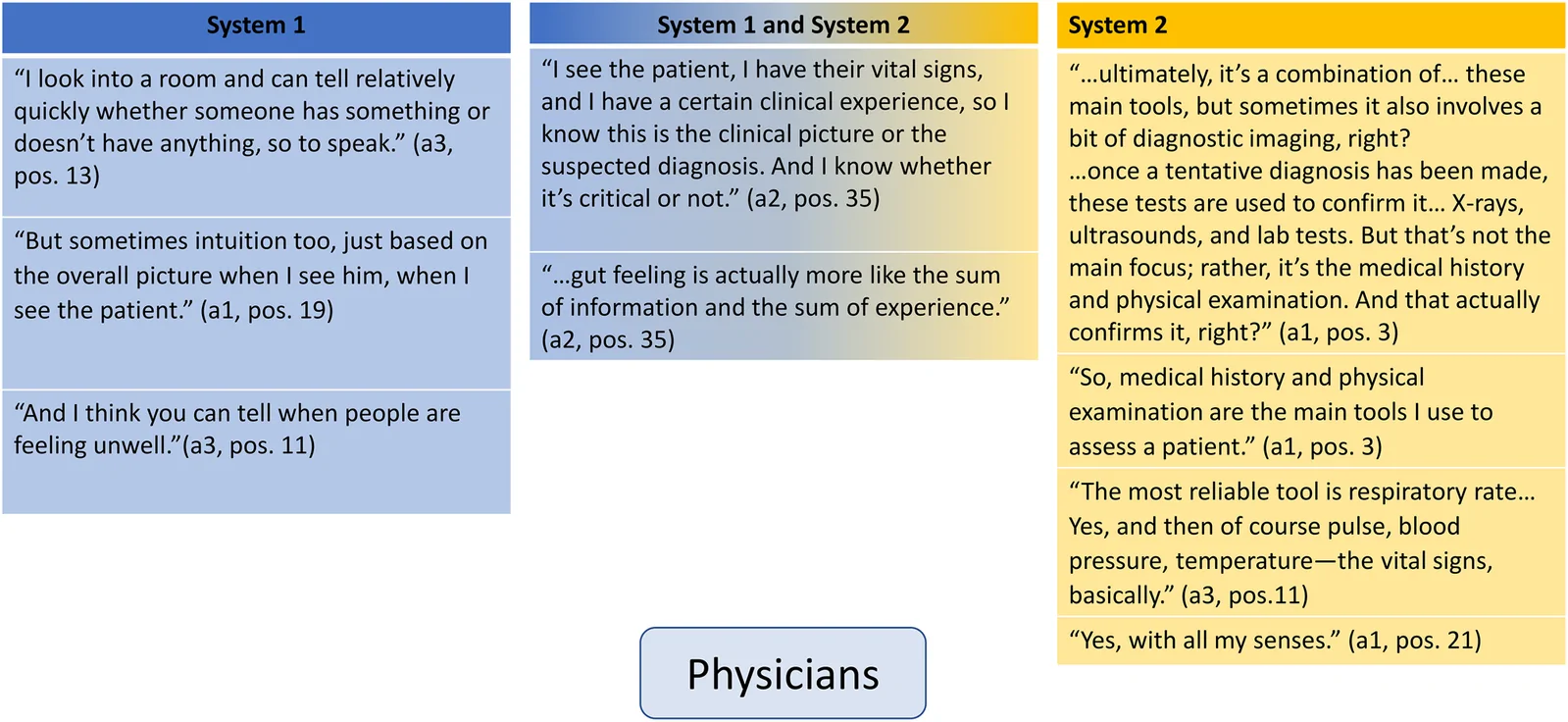
Dual-process Theory in the individual CR. (blue: system 1; orange: system 2; own illustration). (**a**) individual CR of nurses. ABCDE is an acronym (airway, Breathing, Circulation, Disability, environment) for guided assessment. SAMPLE is an acronym (signs and symptoms, allergies, medications, past medical history, last oral intake, and events leading up to the incident) for guided anamnesis. (**b**) individual CR of physicians

#### Relevant factors in the CA and cognitive actions corresponding individual-cognitive CR

Both professional groups cited vital signs and diagnostic imaging as key tools for objectively assessing symptoms and establishing a basis for prioritizing interventions during the initial care of emergency patients. For both physicians and nurses, the integration of these objective measurements with the patient’s medical history and clinical evaluation was main point. In particular, the overall clinical impression and the accompanying intuition were central, experience-based heuristics that can, even independently, lead to increased monitoring or expanded diagnostics. Intuition and overall clinical impression were discussed as important decision-making aids in relation to formulating hypotheses during the initial phase of patient care, but also to make diagnostic decisions, particularly concerning the performance of further diagnostic investigations or the initiation of readily accessible monitoring. Describing the components of overall clinical impression, the both professional groups identified similar factors: facial expressions and posture, skin color, respiration, alertness, and behavioral abnormalities such as agitation- but nurses described them more detailed.

In both professions, elements of system 1 and system 2 of the dual processing model became apparent (Fig. 4). Physicians in the sample emphasized reflexivity through systematic questioning and discussed their intuition (System 1; Fig. 4a) as a process linking contextual factors and experiential knowledge, whereas interviewed nurses placed greater emphasis on the implicit experiential knowledge, describing it as a “gut feeling” (system 1; Fig. 4b).

Differences between the professions were also evident in the perception of the relevance of the physical examination, which physicians viewed as an important basis for assessment, whereas the nurses` focus was on clinical observation. The relevance of the third-party medical history and social background obtained from family members was also highlighted by both professions, but nurses reported to incorporate these environmental data directly into the CR process.

### Social-interprofessional level of CR regarding IPC in the in the daily routine of the ED

#### IPC in the ED

Both professional groups underlined the low threshold for consulting physicians based on nurses´ assessments, who performed triage and initial diagnostics and decided on further monitoring and direct notification of physicians. Criteria for decision-making and for escalating interventions were named and clear team procedures were described. Particularly with critically ill patients, direct face-to-face communication was described and processes of diagnosis and measures of physicians and nurses often occurred in parallel. Handoffs from emergency medical services personnel were handled jointly.

Physicians in the sample stated that trust in the assessments of nurse colleagues depended on the level of the nurses´ work experiences and the sense of familiarity within the team. Overestimating or underestimating the urgency of treatment was identified as a risk and addressed through team coordination.

#### Positive IPC: Prerequisites for functioning IPC in the ED

IPC was perceived as positive above all when the principles of good collaboration were met. Participants described IPC as positive primarily when collaboration took place on an equal footing and flat hierarchies existed, the division of labor was practiced, and nurses were actively involved in decision-making. In the sample, physicians reported taking on nurses´ tasks in acute situations, combined with role flexibility and pragmatism, whereas nurses described well-established interprofessional processes as very positive.

#### Negative IPC: Barriers for functioning IPC in the ED

Participants portrayed IPC as negative in particular when the principles of collaboration were not adhered to, but structural problems also had a negative impact on IPC. Both professional groups described problems of this nature with individuals and certain medical specialties. Nursing staff also addressed interprofessional issues related to referrals to specialized disciplines. In the sample, nurses criticized significant differences in the availability and responsiveness of physicians. During routine daily working times, a reliable presence and IPC were reported; however, when emergency patients were treated by the on-call physicians of various clinics, delays were repeatedly cited. Legal uncertainties regarding telephone orders and lack of availability were also mentioned and were particularly relevant in cases of low trust.

#### Team spirit as part of IPC and social-interprofessional level of CR

Both professions emphasized a well-coordinated core team in the ED characterized by a high degree of familiarity, clear expectations, and rapid, task-oriented interaction.

In the sample, nurses demonstrated a broad understanding of the team (including medical assistants, cleaning staff, emergency medical services, radiology staff), whereas physicians restricted their definition of ‘team’ to the core physician-nurses team.

#### Communication as part of IPC and social-interprofessional level of CR: Content, technical language and tone

Communication content was mostly identical across professional groups. Some nurses described asking for physicians´ assessments out of interest, some reported they shared their assessment with physicians to guide them in order to improve the patient’s treatment.

In the sample, physicians described a didactic guidance for nurses as an aim when sharing their thoughts on their CA and CR, with the goal of improving the nurses´ initial CA and CR.

**Fig. 5:**
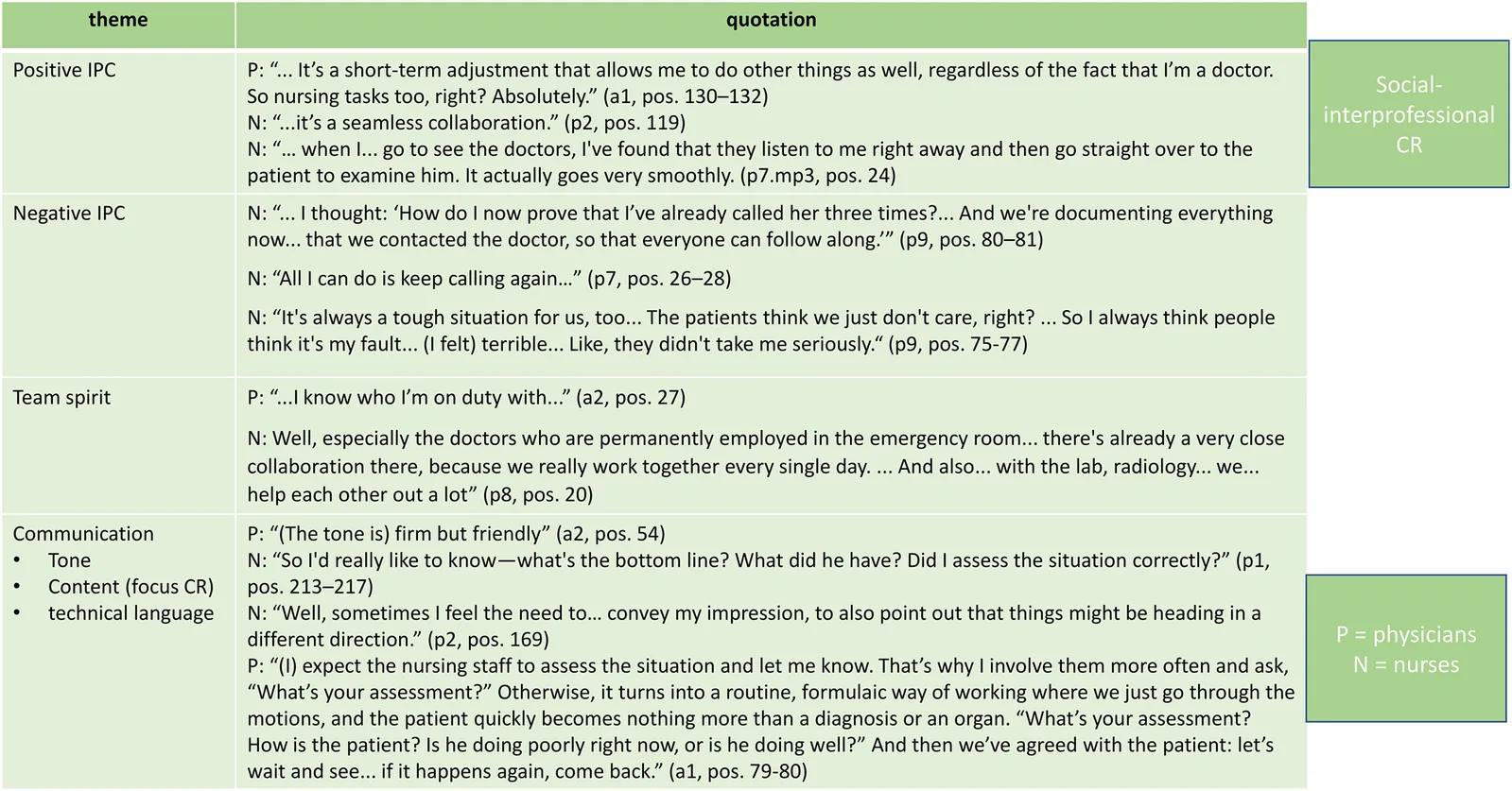
Social-interprofessional CR: themes and quotations

In both professional groups, adapting technical language to the listener was discussed. There were few reported barriers within the interprofessional core team of the ED, communication problems tended to arise with external physicians. Physicians in the sample named some targeted adaptation of terms; nurses perceived fewer differences, some terms were actively asked about or looked up later. The tone of interaction was described as “firm but friendly” (a2, item 54). Figure 5 displays the key points analyzed and corresponding quotations within the social-interprofessional CR in the sample.

### Organizational-systemic influencing factors

#### Influence on the individual-cognitive CR: Working experience in the ED

Interviewees considered working experience an important prerequisite for working in the ED. But the description of the initial CA and CR of the interviewees varied based on working experience in the ED. In the sample, nurses with 1–5 years of working experience in the ED stated strongly guided assessment tools as the MTS or the ABCDE algorithm (for Airway, Breathing, Circulation, Disability, Environment) for as an important aid in their assessment, whereas nurses with more years of working experience in the ED emphasized the importance of intuition, the relevance of the overall impression, and the importance of an independent, unbiased assessment. Physicians´ trust in the nurses´ assessment also was described by the interviewed physicians as depending on the working experience of the nurses.

#### Influence on social-interprofessional CR: Interprofessional debriefings and team-timeouts

Planned and systematic interprofessional debriefings to discuss difficult situations in the ED

were generally recognized as important by all interviewees. Physicians cited clearly established structures; from the nurses’ perspective, contradictory statements were made reporting well functioning planned and structured debriefings following difficult situations, as well as unmet needs.

#### Influence on social-interprofessional CR: Region and size of the ED

Long waiting times due to a lack of physician presence were attributed to high workloads for physicians and structural problems in smaller teams (rural regions). On the other hand, in the sample, nurses in larger EDs interpreted a lack of availability and presence as “not being taken seriously” (p9, lines 75–77) and described a certain helplessness.

With regard to the location and size of the ED, differences in processes were also evident. For instance, routine procedures in trauma room management were central at the tertiary care facility, whereas at the primary care facility, the proactive nursing initiative was emphasized.

#### Influence on social-interprofessional CR: Digitization

Digitization in medical documentation, as an important medium for collaboration, was generally perceived as positive by all professional groups and age groups. It was emphasized that digitally documented orders and measures provide security and traceability and improve the flow of information across locations and sectors. Scheduling views and digital task lists eliminated the need for follow-up inquiries and thus optimized time resources. The main negative aspect described was an increased workload due to poor usability.

**Fig. 6:**
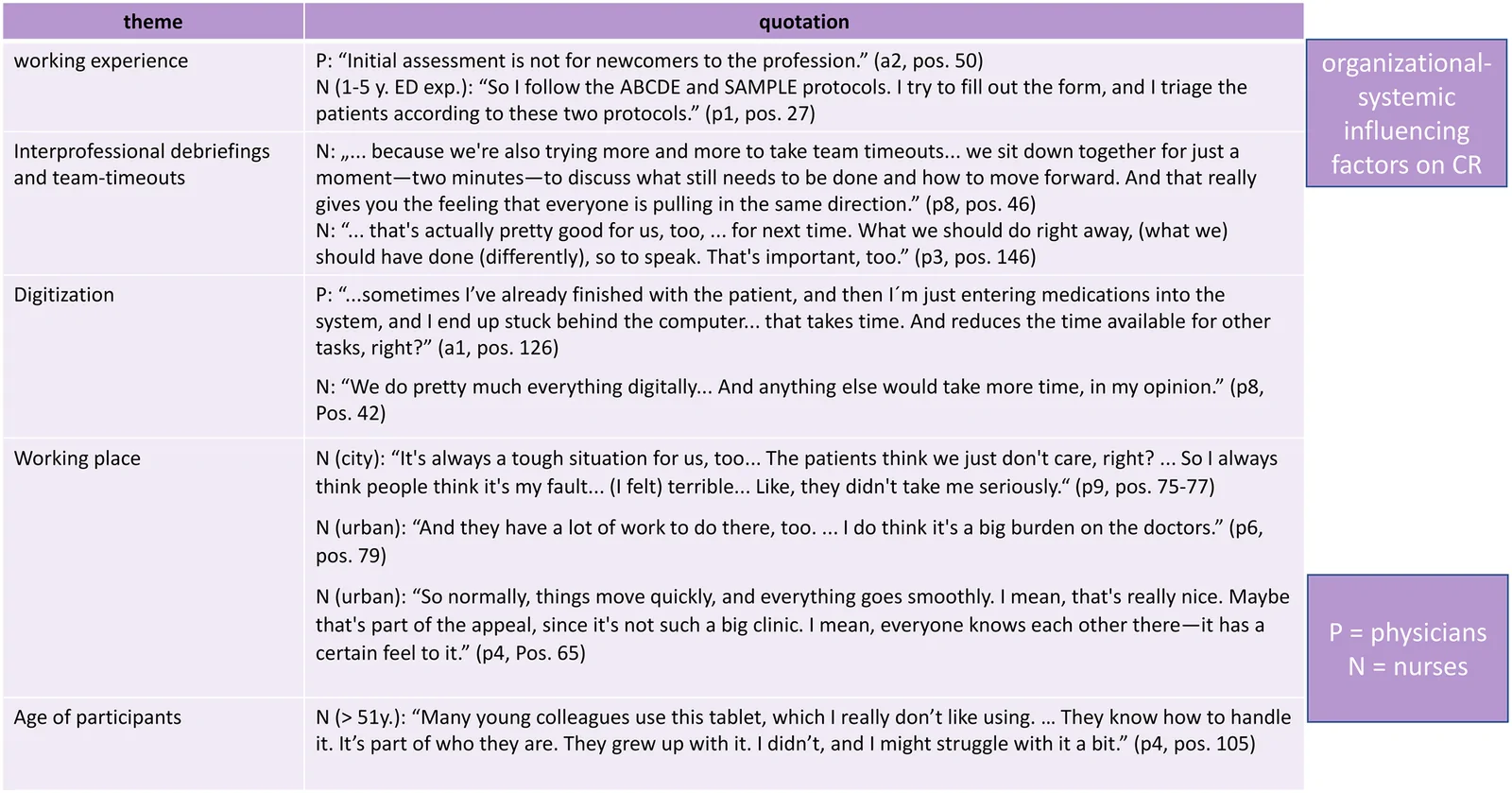
Organizational-systemic influencing factors on CR: themes and quotations. ABCDE is an acronym (airway, Breathing, Circulation, Disability, environment) for guided assessment. SAMPLE is an acronym (signs and symptoms, allergies, medications, past medical history, last oral intake, and events leading up to the incident) for guided anamnesis

In the sample, differences in the perception of digital applications suggested also age-dependence. The majority of participants did not perceive a reduction in direct communication due to increasing digitization. Figure 6 summarizes the most important points discussed in the interviews regarding organizational-systemic influencing factors on CR.

### Prerequisites and suggestions for improvement

Participants identified approaches for improving IPC in ED at various levels.

- Sufficient clinical working experience
- Personal factors: stress resilience, flexibility, teamwork and communication skills, curiosity, and the ability to remain calm and make quick decisions
- Structural level: changes in the triage process, improvement of the nursing staff-to-patient ratio, parallel medical and nursing initial assessments, spatial changes, interprofessional and interdisciplinary continuing education (trauma room training, simulations, specific training courses), structured practical guidance for young nursing professionals, system improvements (usability)

## Discussion

This pilot study analyzed CR as a multilevel process at the individual-cognitive, social-interprofessional, and organizational-systemic levels.

In the CA, as part of the CR, there were many overlaps between the group of physicians and the nursing staff. The differences in the weighting of some items in the CA are easy to understand, especially since, even during education, nurses tend to focus more on observing patients than on physical examination. This is also evident in the description of the aspects that sum up the overall impression of patients: facial expressions and posture, skin color, respiration, alertness, and behavioral abnormalities such as agitation. Both professional groups identified similar factors: facial expressions and posture, skin color, respiration, alertness, and behavioral abnormalities such as agitation, but nurses in the sample described them more detailed.

The Dual Process Theory as a methodology was not chosen as a prescriptive model for how clinical decisions should be made in the ED, but rather as a theoretical framework designed to raise awareness, to structure the various forms of cognitive processes such as CA, information processing, drawing conclusions, making diagnoses, or determining the need for treatment, observed in the interviews, and, in particular, to highlight the interplay between experience-based and analytical-systematic thought processes.

The results of individual-cognitive CR show that, in the present sample, both professions utilize elements of both systems of the Dual-process Theory [16, 19]. Differences between the professional groups of physicians and nurses in the sample were evident in their differing emphasis on the importance of the physical examination and in greater reflection on their own actions and thought processes. Physicians reflected more on the cognitive process itself and evaluated intuition as a process that combines contextual factors and experiential knowledge, while nurses in the sample emphasized the relevance of fixed criteria (System 2), clinical experience in general, related to the development of “gut feelings” (System 1). Experienced nurses in the sample emphasized that the fixed criteria of the MTS alone are insufficient for assessing patients. It has been demonstrated that nurses often go beyond triage algorithms in their initial assessment to assess patients holistically [19] and that CA in the ED is not possible based solely on triage systems [6]. While the MTS provides a structured framework for determining treatment urgency, the CA and CR process described by the nurse participants extended beyond the assignment of a triage category. Participants described the integration of presenting symptoms, clinical observations, prior experience, contextual information and patient and relative communication, representing the application of fixed criteria and intuition. The physicians interviewed also described clearly defined thought processes, such as focusing on a structured medical history and physical examination, the great importance of vital signs, and the supportive role of blood tests and diagnostic imaging, as standardized through Standard Operating Procedures (SOPs).

These results match to the statements of Pellacia et al., that a harmonious interaction between Systems 1 and 2 could be the crucial key function of CR in the ED setting [17]. Furthermore, the results of this study also appear to align with current findings on CR in the ED, which show that clinical reasoning is a core competency not only for physicians [14, 15], but also for nurses [4, 10] and that registered ED nurses utilize both System 1 and System 2 just as physicians do and show similar cognitive processes am [19].

It is not surprising that both systems play an important role in emergency medicine. After all, the findings should not be interpreted as suggesting that intuitive judgement can replace systematic CA. In the ED, a symptom-oriented approach is applied in order to accurately identify clinical causes and to ensure appropriate and timely treatment. Although it is important to be able to act quickly, it is also essential to ensure that best possible patient safety is ensured. To achieve this, the approach has to be based on established standards as structured assessment and clinical standards to minimize the risk of overlooking a rare yet serious condition. Thus, CA, CR, determination of diagnosis and decision on treatment cannot be based solely on System 1. This is also confirmed by the statements of the participants interviewed in this study. Rather, the findings illustrate the complementary nature of the two modes of cognitive processing. In emergency medicine, rapid pattern recognition and experience-based judgements may initiate or guide CA, while analytical reasoning can subsequently verify, challenge, or refine these impressions. Conversely, systematic approaches may generate information that triggers or modifies an intuitive judgement. Thus, the two systems should not be understood as mutually exclusive or hierarchically ordered modes of CR.

Since both nurses and doctors exhibit a combination of elements from Systems 1 and 2 in their individual CR, this commonality—as well as the differences in approach—offers a valuable opportunity to place a greater emphasis on the IPC between the two professional groups far beyond triage. Clinical impressions may be communicated, discussed or complemented by another professional perspective. The exchange, challenge and integration of different perspectives could validate individual clinical impressions through additional information and more deliberate reasoning, improving IPC but also patient care. The nursing role involves establishing a relationship with patients through which additional confidential information is often received and can be incorporated into the treatment process, contributing to stabilization in acute situations using a holistic approach.

To analyzed CR on the social-interprofessional level, IPC in the ED and prerequisites for and barriers against functioning IPC in the ED were extracted. The participants in this sample overwhelmingly viewed IPC within their core team positively and expressed known key factors [20] as reasons for successful and positively perceived collaboration. They described collaboration on an equal footing, with flat hierarchies, knowledge of one’s counterparts, mutual trust, clear expectations, respect, and appreciation as fostering a positive team spirit. Sharing one’s own assessment and involvement in decision-making—as part of active inclusion of nurses into decision-making processes of physicians—was also perceived positively. Communication as part of IPC and social-interprofessional level of CR showed not relevant differences in content or technical language within the core team of the ED. In the sample, nurses shared their own assessments both out of curiosity and to guide physicians in the right direction; physicians, on the other hand, tended to describe situations in which they provided guidance to improve the nurses´ assessment of patients and their treatment.

Regarding building up professional roles, nurses and physicians are socialized to during their education, adopt a disciplinary perspective and strictly delineated professional areas of responsibility [30] and perceive different core tasks as central to their roles; however, various overlaps in their scope of practice also require role flexibility [31]. This aspect is also reflected in the results of the present study.

The results of this study show that IPC is particularly successful when the principles of collaboration are adhered to [20] and that obstacles primarily arise from violations of these principles. Person-related hierarchical role conflicts and unprofessional behavior in the clinical context are reported as relatively common in the literature [32], disrupt IPC, and lead to risks for patients [33, 34].

IPC improves significantly through knowledge of the expertise, roles, and responsibilities of the involved stakeholders and the accompanying understanding of the other profession [30, 35]. The interdisciplinary and interprofessional continuing education and simulations desired by the participants in the present sample could address this aspect. In this context, the expansion of interprofessional competencies, such as interprofessional communication, appears particularly relevant [35–37]. Various interventions offer a potential approach to improving IPC and patient care [7, 38]. Such interventions often focus on interprofessional continuing education and structural changes (interprofessional rounds, meetings, checklists), but also incorporate sociocultural perspectives (interprofessional communication, external activities) [7, 38].

The two theoretical frameworks used in this study address complementary dimensions of CR and IPC. The Dual Process Theory provides a framework for understanding how clinical information is processed in the individual, encompassing both rapid, experience-based judgements and more deliberate analytical reasoning. D´Amours’ framework, in contrast, focuses on how professionals work together and integrate their respective perspectives within IPC. In this way, beyond the individual interplay of rapid, intuitive, and analytical, confident assessments within individual CR, the exchange of assessments between the two professions could also be understood as a mechanism that verifies or supplements intuitive assessments or translates them into a systematic decision-making process. Thus, an intuitive assessment of a member of the ED team based on System 1 could serve as a possible starting point for an iterative IPC process that incorporates elements of both systems. Cognitive decision-making processes, as described in the Dual-Process Theory, should not be considered in isolation within a single individual; rather, the exchange of information among the professional groups that assess patients and make decisions should be taken into account. The perspectives of the Dual-Process Theory and the model of good IPC should be considered together and closely intertwined, because in the ED, CR is not only an individual, isolated, cognitive process, but takes place within an interprofessional team. Thus, the interaction between different modes of clinical processing may be embedded within the processes of IPC.

Furthermore, the stakeholders involved interact in an environment that entails a range of organizational and structural constraints and, in some cases, uncontrollable influences, which further increases the complexity of the relationships among the professionals [16, 19, 20]. A barrier to interprofessional models of CR is a lack of time, which underscores, that structural changes are important to enable better-coordinated IPC [24]. Nurses in the present sample complained about the lack of availability and presence of physicians during on-call hours. Interestingly, the interpretation of the resulting waiting times differed between small and larger teams, which could be explained by the greater familiarity within smaller teams. This could address a structural-organizational issues of the ED during night shifts that negatively impact IPC, but also emphasizes the relevance of team spirit in the ED. In many EDs, emergency nurses are the permanent members of the ED team, often have significantly more professional experience than physicians and form the core of the emergency team. In contrast, physicians are more often assigned to EDs for only a brief period as part of a rotation during their specialty training. This organizational structure influences the team’s dynamics as well as the roles of its members. In the sample studied, only senior physicians with completed specialty training who were employed and assigned to the ED on a long-term basis were interviewed.

An Influence on the individual-cognitive level of CR can be seen in the working experience in the ED. Less experienced nurses in the sample reported relying on fixed algorithms like the MTS and the ABCDE algorithm [39] which could be assigned to System 2 of the Dual process Theory, which can be helpful [19], as the cognitive processes based on System 1 are significantly determined by the decision-maker’s experience and are even less developed in less experienced individuals [16]. Whereas nurses with more years of working experience in the ED in the sample emphasized the importance of intuition, the relevance of the overall impression, and the importance of an independent, unbiased assessment. Also, physicians´ trust in the nurses´ assessment was described by the interviewed physicians as depending on the working experience of the nurses. The relevance of clinical experience for the quality of initial CA and CR, as emphasized by the participants, is well documented [7, 16, 19, 23] and represents a sensible prerequisite for working in the ED.

Regarding the interprofessional communication as part of the IPC, planned and systematic, organizationally implemented interprofessional debriefings and team-timeouts were generally recognized as important by all interviewees in the sample.

The digitization of documentation was also examined as a possible influencing factor on interprofessional communication and social-interprofessional CR. Although generally perceived as positive by all professional groups and age groups and in the sample, some interviewees criticized increased workload due to poor usability. An age-dependence in the differences in the perception of digital applications can be suggested in the data, but is not completely trustworthy due to the limited number of participants.

As a way to strengthen social-interprofessional CR, further Organizational-systemic adjustments would be necessary. Concepts of collaborative interprofessional decision-making in the ED and care provided on an equal footing offer enormous advantages, but require not only continuing education but also structural adjustments [22, 23, 40, 41].

### Limitations

The small number of participants and the composition of the sample is also a limiting factor; of a total of 13 interviews, only three were conducted with physicians, limiting comparability between professional groups and reducing the transferability of the results. Even though no significant new aspects were found in the present sample after the third analyzed interview with physicians, saturation cannot be reliably assumed given this small number of interviews, which limits the explanatory power of the analyses regarding the physicians. Findings related to specifically to physicians should therefore be interpreted as exploratory rather than trustworthy. Nevertheless, the physicians´ perspectives contributed important complementary insights.

Consequently, the findings reflect primarily the perspective of nursing professionals, with limited input from the perspective of physicians.

There are possible biases regarding concepts, because no clarification of the concepts and terminology took place before the interviews began. It was assumed that the interviewees, as specialists in the practical work in the emergency room, would be able to correctly classify terms like “triage.” To reduce misunderstandings due to excessive complexity or abstraction, many terms used for the analysis were not explicitly named in the interview questions. For example, the question was not about “interprofessional communication,” but rather specifically about when, how, and on which topics contact was made with the respective other professional group. Nevertheless, there is a risk, that not all participants understood the concepts of triage or assessment at the same way and to prevent confusion, these terms could have been discussed beforehand to create a clearer starting point to avoid differing views on the definitions.

The interviewees may have been influenced by social desirability bias, as they may have portrayed interprofessional collaboration within their own team more positively and been more reluctant to discuss negative aspects. However, in the present study, there was no power imbalance between the interviewers and the interviewees. None of the interviewers were employed by the same hospital group at the time of the interviews, and, of course, the individual statements made during the interviews were not made public to other interviewees or anyone else.

The substantial variation in interview duration may have resulted in differences in depth and breadth of das obtained across participants. Shorter interviews may have provided less opportunity for participants to elaborate on their experiences compared with longer interviews. However, given the open-ended nature of the questions, interview duration was not considered a direct indicator of data quality and richness. All relevant topics were addressed in all interviews

The study reveals a lack of data triangulation because only one type of data and method was used. Nevertheless, the authors tried to maintain investigator triangulation through a research team with diverse backgrounds (physician and nurses) to reduce biases. Data was analyzed independently by two members of the research group who then met to discuss their interpretations, identifying areas of agreement and disagreement. In case of the need for new subcategories, the data was revised.

Since the results are based on a small and selective sample from a single provider, transferability is limited. Furthermore, a self-selection effect cannot be ruled out, as individuals with a fundamental interest in interprofessional collaboration may have been more willing to participate in the study.

The interviewees tended to be older and more experienced; while this highlights the relevance of experience to the clinical assessment of emergency patients, it also suggests that younger individuals—who might have had different experiences—could be underrepresented. Furthermore, despite the consensus-based coding process, the influence of individual researchers’ subjective interpretations cannot be entirely ruled out.

The duration of interviews varied between 25 and 90 minutes. Although this variation was inherent to the use of open-ended questions, the considerable variation in the interview duration may have influenced the deapth and breadth of the data generated and should therefore be considered a limitation.

Due to the given limitations this study is understood as a pilot study that could inform a future larger-scale study.

## Conclusion

This pilot study provides interesting insights CR in the ED and suggest an analysis of CR on different levels and could inform a larger-scale study. The results demonstrate that the approaches of physicians and nurses within the individual-cognitive CR involve both intuitive and analytical processes, influenced primarily by clinical experience in the emergency field. The slightly different approaches of nurses and physicians are complementing each other when IPC is effective. On the social-interprofessional level of CR, well-coordinated teams that adhere to rules for effective IPC, resulting in respectful, equal-status interaction show few communication barriers and are associated with high levels of satisfaction in IPC. The different cognitive processes of CR should not be considered in isolation within an individual but rather should be embedded within the concepts of IPC as both levels of CR are intertwined. Organizational-systemic influencing factors can be understood as prerequisites for successful CR. In this way, the results offer approaches for improvement of CR in ED: On the individual-cognitive level of CR, working experience in the ED setting seems to be important; on the social-interprofessional level of CR, resources for example in nightshifts, structured debriefings and team-timeouts, but also familiarity of the team, flat hierarchies and mutual respect were identified as relevant factors in this study.

## Data Availability

Due to the inability to fully anonymize the collected data (only pseudonymization is possible), the datasets cannot be made fully publicly available because individual privacy could be compromised. Thus, the datasets generated and analysed during the current study are not publicly available but are available from the corresponding author on reasonable request.

## Abbreviations

CA: Clinical Assessment
CR: Clinical Reasoning
ED: Emergency Department
IPC: Interprofessional Collaboration
MTS: Manchester Triage System
